# Estimation of Unsteady Aerodynamics in the Wake of a Freely Flying European Starling (*Sturnus vulgaris*)

**DOI:** 10.1371/journal.pone.0080086

**Published:** 2013-11-22

**Authors:** Hadar Ben-Gida, Adam Kirchhefer, Zachary J. Taylor, Wayne Bezner-Kerr, Christopher G. Guglielmo, Gregory A. Kopp, Roi Gurka

**Affiliations:** 1 School of Mechanical Engineering, Tel-Aviv University, Tel-Aviv, Israel; 2 Boundary Layer Wind Tunnel Laboratory, Faculty of Engineering, University of Western Ontario, London, Ontario, Canada; 3 Department of Biology, Advanced Facility for Avian Research, University of Western Ontario, London, Ontario, Canada; 4 Department of Mechanical Engineering, Ben-Gurion University of the Negev, Beer-Sheva, Israel; Rensselaer Polytechnic Institute, United States of America

## Abstract

Wing flapping is one of the most widespread propulsion methods found in nature; however, the current understanding of the aerodynamics in bird wakes is incomplete. The role of the unsteady motion in the flow and its contribution to the aerodynamics is still an open question. In the current study, the wake of a freely flying European starling has been investigated using long-duration high-speed Particle Image Velocimetry (PIV) in the near wake. Kinematic analysis of the wings and body of the bird has been performed using additional high-speed cameras that recorded the bird movement simultaneously with the PIV measurements. The wake evolution of four complete wingbeats has been characterized through reconstruction of the time-resolved data, and the aerodynamics in the wake have been analyzed in terms of the streamwise forces acting on the bird. The profile drag from classical aerodynamics was found to be positive during most of the wingbeat cycle, yet kinematic images show that the bird does not decelerate. It is shown that unsteady aerodynamics are necessary to satisfy the drag/thrust balance by approximating the unsteady drag term. These findings may shed light on the flight efficiency of birds by providing a partial answer to how they minimize drag during flapping flight.

## Introduction

Flapping flight is one of the most complex yet widespread propulsion methods found in nature. Although aeronautical technology has advanced remarkably over the past century, flying animals still demonstrate higher efficiency [1]–[3]. One of the key open questions is the role of unsteady fluid motion in the wake of flying animals and its contribution to the forces acting during the downstroke and upstroke [4]. The unsteady flow over small-scale wings has gained significant attention recently both in the study of bird and insect flight as well as to develop advanced aerodynamic models for high-performance micro-aerial vehicles [2]). The goal of the current study is to examine both the steady and the unsteady aerodynamics in the wake of a freely flying bird with a particular focus on the propulsive forces.

Among the first few attempts to describe unsteady aerodynamics [5]–[7], Brown [7] distinguished several patterns of flapping flight and described complex movements of the wings through multiple sets of illustrations for different types of birds. Recently, Brunton and Rowley [8] developed reduced-order models for the unsteady aerodynamic forces on a small wing in response to agile maneuvers and gusts based on the framework suggested by Wagner [5] and Theodorsen [6]. However, they did not manage to augment their model with non-linear stall and separation models, which are important for improving lift modeling (see for example: Henningsson et al. [9]).

According to quasi-steady-state aerodynamic theory, slow-flying vertebrates (e.g., insects) should not be able to generate enough lift to remain aloft [10]. Therefore, unsteady aerodynamic mechanisms to enhance lift production have been proposed. Muijres et al. [11] showed that unsteady aerodynamic mechanisms are used not only by insects but also by larger and heavier fliers. Hubel and Tropea [12] verified Muijres et al. [11] findings by showing that the unsteady effects are not negligible for a goose-sized flapping model. Thus far, the main purpose of investigating unsteady aerodynamic mechanisms has been to understand their ability to enhance lift generation. However, it remains relatively unknown how unsteady aerodynamics participate in the drag and thrust balance of flapping flight.

The complex unsteady features of flapping flight introduce challenges to any realistic aerodynamic analysis. One of the first attempts to incorporate realistic wake structure in an aerodynamic model of bird flight was by Rayner [13]–[15] who proposed that each wingbeat was only aerodynamically active during the downstroke. As part of the wake structure, starting and ending vortices were suggested to be produced at the beginning and at the end of the downstroke phase, respectively. These vortices are connected by a pair of trailing vortices shed from the wingtips [13]. Therefore, at a certain distance downstream, the wake was assumed to be composed of a series of vortex rings referred to as ‘elliptical loops’ [14] which were conceptually related to the bird’s wingspan and the circulation dictated by the force requirements (lift, profile drag and parasitic drag) of the bird. However, Rayner’s model did not match later experimental observations. Spedding [16] performed measurements of vortex circulation in a jackdaw (*Corvus monedula*) wake and indicated that approximately half of the required momentum for weight support was present for jackdaw flight. As a consequence, the ‘wake momentum paradox’ arose. It was concluded that the discrepancy could be a result of unidentified complexities in the wake structure; i.e., Rayner’s model was too idealized. Spedding [17] performed experiments with the same apparatus as for the jackdaw on kestrel (*Falco tinnunculus*) flight at moderate (*U_∞_* = 7 m/s) speeds and observed a distinctly different wake topology. He found that, instead of discrete loops separated by aerodynamically inactive upstrokes, two continuous undulating vortex tubes exist in the wake; i.e., the upstrokes were also aerodynamically active. The measured circulation of the shed vortices was similar during the downstroke and upstroke and it was adequate for supporting the weight of the bird. In addition to the lift force it was found that generating net thrust occurred through varying the wing geometry – not through varying the circulation – implying that the wing motion is important.

The concept of different wake topologies prompted analyses of vortex gaits [18]–[20]. Rayner [18] characterized the vortex gait selection for flying birds and stated that the choice between the different vortex gaits is determined by flight speed and wing morphology. Spedding et al. [20] investigated the wake structure behind a thrush nightingale (*Luscinia luscinia*). They concluded that the structure of the wake downstream, far from the body (roughly 17 chord lengths), varies gradually from an approximately elliptical vortex wake to a continuous trailing vortex wake. Therefore, the wake is comprised neither of a series of elliptical vortex loops, nor a pair of continuous trailing vortices, but is a combination of both. Eventually, the ‘wake momentum paradox’ was addressed, within the bounds of experimental uncertainty, through the high spatial resolution available in PIV as well as a detailed accounting procedure for the calculation of circulation in the wake [20]. However, the relationship between specific wake topology and the propulsive aerodynamics of bird flight remains an open question.

The vorticity structure in the wake of a flying bird, similar to the distribution of vorticity in any wake, is dependent on the boundary conditions. Consequently, the kinematics of the wings have been investigated in the literature with the goal of either using the wing motion to predict forces or associating the wake topology with the motion of the wings. Two wingbeat kinematics of a thrush nightingale [21], and of two individual robins [22], were quantified in order to relate them to their vortex wakes. However, the kinematic variations with flight speed occurred only during the upstroke period where the wing folding and the wingbeat frequency were observed to vary. In addition to the wingbeat kinematics, the streamwise distance between the bird and the measurement location has been shown to be of importance when drawing conclusions about the aerodynamics related to the wake structure. Hedenström et al. [22] investigated wakes behind European robins (*Erithacus rubecula*) and found that they resemble the thrush nightingale wake [20]. It was argued that the circulations of the wakes were similar because the measurements were in the far wake significantly downstream of the bird [4]. Studying both the near and far wake of Pallas’ long tongued bat (*Glossophaga soricina*), Johansson et al. [23] concluded that measurements in the far wake might lead to misinterpretation of the wake topology. This misinterpretation occurs because the near wake is more readily tied to the generating wing kinematics and, thus, contains details of vortex structures that could easily be missed in the far wake [23].

The contribution of unsteady aerodynamics to force calculations on flying birds, which results mainly from the wing’s motion, has not been addressed rigorously in the past [4]. This may be attributed to the complexity in modeling unsteady effects and predicting their role. Furthermore, even within the framework of a model describing unsteady effects, it is only recently that experimental systems such as PIV are able to resolve both temporal and spatial scales. Rayner et al. [24] performed measurements on starlings in undulating flight in a wind tunnel and showed that the geometry of the flight path depends upon wingbeat kinematics, and that neither the flapping nor the gliding phases of flight occur at constant speed or at constant angle to the horizontal. The bird gains both kinetic and potential energy during the flapping phases making it difficult to model. Rayner et al. [24] indicate that such speed variation can provide significant savings in mechanical power in both bounding and undulating flight. Recently, high-speed PIV systems have become available for animal flight research to analyze the wake structure at high temporal resolution [25], [26]. In these studies, the wake is sampled using PIV images taken at a typical frequency of 200 Hz in a transverse plane (vertical spanwise) referred to as the Trefftz plane [27]. The three-dimensional wake is assembled by identifying coherent streamwise structures, such as the tip vortices [28], and the time varying flight forces have been estimated based on this method [29]–[32]. However, the focus in such studies has been on the lift force and not on the relation between drag and thrust. It is also noteworthy that PIV data in the Trefftz plane have substantial measurement uncertainty in the estimated velocity and velocity gradients due to the set-up complexity and the nature of the PIV technique when performing flow measurements with a strong out of plane velocity component [27]. Therefore, conclusions drawn based on these measurements should be utilized carefully. To date, there is no available volumetric technique capable of performing full three-dimensional measurements of high speed flows in air. The existing techniques such as StereoPIV provide three velocity components but not gradients. In addition, the accuracy level of this technique in reconstructing the third dimension is not high [33], [34]. Therefore, the current models and quantitative estimations of forces behind the wake in the Trefftz plane are subject to relatively large errors [27].

Currently, many of the aerodynamic models for birds are based on fixed wings in steady flow [35]. Recently, there is a growing interest in using realistic models [2], [12] because many engineering applications such as UAV’s are designed based on avian aerodynamics and the performance of birds in different flight modes [36]. The current study addresses the near wake variations behind a freely flying bird in time and space with a particular focus on the unsteady aerodynamics that results from the flapping wing. The change of velocity with time is the key parameter that marks the unsteady effects and its variation is examined in terms of the drag – thrust balance.

## Experimental Setup

### Wind Tunnel

The experiments were performed in the closed-loop hypobaric climatic wind tunnel at the Advanced Facility for Avian Research (AFAR) at the University of Western Ontario. The octagonal test section of the wind tunnel has a cross-sectional area of 1.2 m^2^, preceded by a 2.5∶1 contraction. The width, height and length of the test section are 1.5 m, 1 m, and 2 m, respectively. It is enclosed in a hypobaric chamber allowing for testing at high altitude conditions by controlling pressure, temperature and humidity in the test section. An open jet exists between the downstream end of the test section and the diffuser for the purpose of introducing the live bird into the wind tunnel during the experiments. The turbulence intensity is lower than 0.3% at the location where the measurements were taken. A fine net was placed at the upstream end of the test section to prevent the bird from entering the contraction, which was not observed to alter the turbulence significantly. The flight conditions were at atmospheric pressure, a temperature of 15°C, and relative humidity of 80%.

### The Bird - European Starling

**Figure 1:**
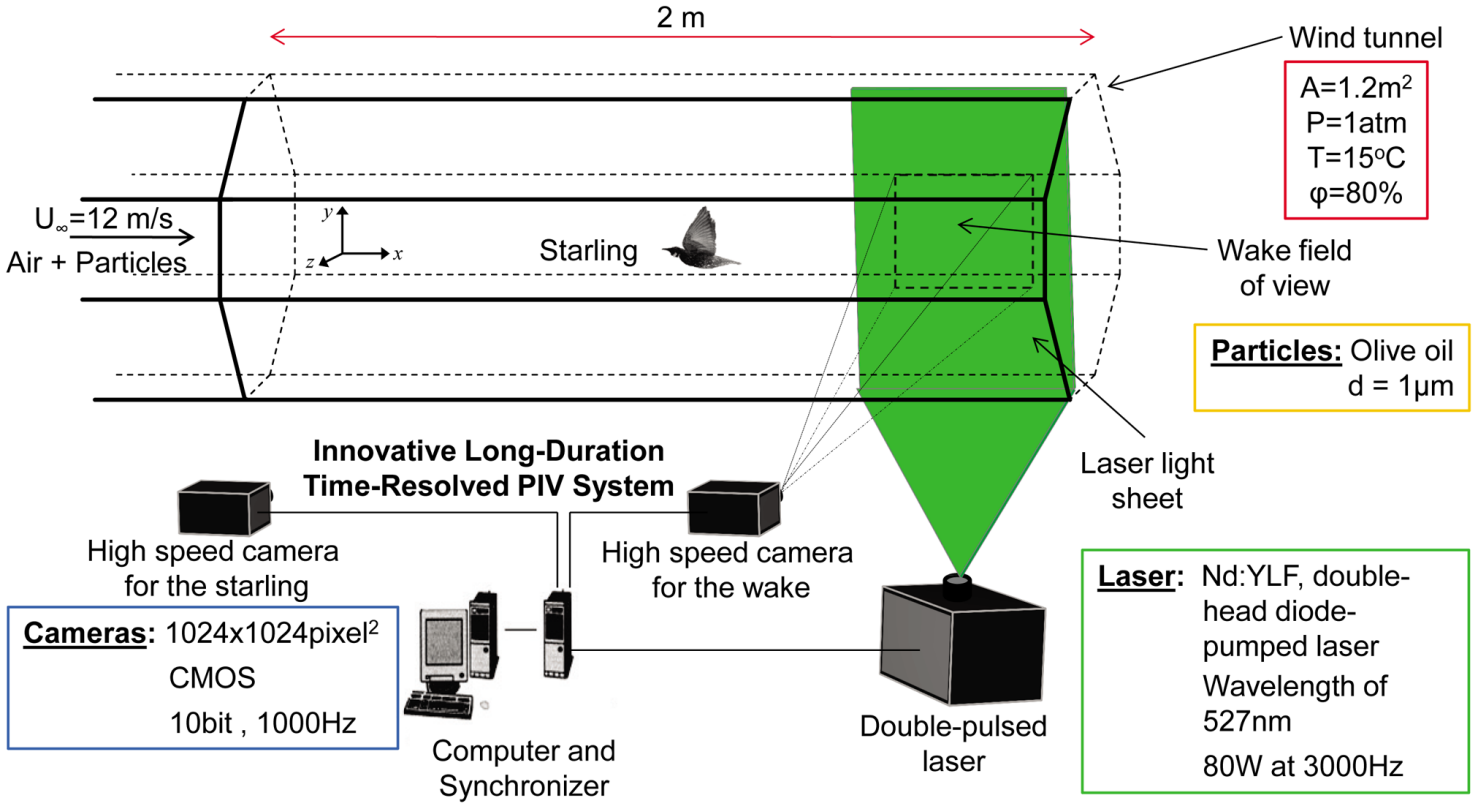
Illustrative scheme of the avian wind tunnel and the experimental setup system.

The wake measurements (as illustrated in Figure 1) were taken from a European starling that had been trained to fly in the AFAR wind tunnel. The bird’s wings had an average chord, *c*, of 6 cm, a maximum wingspan of *b* = 38.2 cm (*b*_semi_ = 19.1 cm) and an aspect ratio (wingspan squared divided by the wings’ lifting area), *AR*, of 6.4. A typical cruising speed of *U*_∞_ = 12 m/s was chosen for the experiments based on the comfort of the starling and its ability to fly for prolonged periods of time during the testing. The wingbeat frequency, *f* (inverse of the period, *T*, of each wingbeat), was 13.3 Hz on average, and the average peak-to-peak wingtip vertical amplitude, *A*, was 28 cm. These quantities correspond to a chord-based Reynolds number of 4.8·10^4^, a Strouhal number, *St* = *Af*/*U*_∞_ = 0.30, and a reduced frequency, *k* = *πfc*/*U*_∞_ = 0.20. At the time the experiments were performed the bird had a mass of 78 g and a lateral body width of 4 cm.

Due to the powerful laser operating within a few chord lengths of the bird’s tail, two precautions were taken to ensure the bird’s safety. Goggles made of a flexible and optically dense polymer material (Yamamoto Cogaku Co YL 600) were designed to protect the bird’s vision as well as to reduce the potential of the light sheet frightening the bird. After an accommodation period of 20 minutes in a cage, the bird would fly normally in the wind tunnel while wearing the goggles. In addition, for preventing direct contact between the bird and the light sheet, a collection of optoisolators operated by six infrared transceivers were integrated into the PIV system. The function of the optoisolators was to trigger the laser only when the bird was in a desired position upstream of the PIV field of view thus ensuring that the bird was in a position where it was not in danger of being hit by the laser. All animal care and procedures were approved by the University of Western Ontario Animal Use Sub-Committee (protocols 2006–011, 2010–216). Further details pertaining to the experiments can be found in Kirchhefer et al. [37].

### Flow Velocity and Kinematic Measurements

Flow measurements were taken using the long-duration time-resolved PIV system developed by Taylor et al. [38]. Olive oil particles, 1 µm in size [39] were introduced into the wind tunnel using a Laskin nozzle from the downstream end of the test section so that it did not cause a disturbance to the flow in the test section or to the bird. The PIV system consists of an 80 W double-head diode-pumped Q-switched Nd:YLF laser at a wavelength of 527 nm and two CMOS cameras (Photron FASTCAM-1024PCI) with spatial resolution of 1024×1024 pixel^2^ at a rate of 1000 Hz. The PIV system is capable of acquiring image pairs at 500 Hz using the two cameras for 20 minutes continuously. In the current experiments, one camera was used to record the bird kinematics during the wingbeat and the other was used for PIV measurements in the wake. The PIV camera’s field of view was approximately 12×12 cm^2^ in size, or 2*c* by 2*c*. Vector fields were computed by OpenPIV [38] using 32×32 pixel^2^ interrogation windows with 50% overlap, giving a spatial resolution of 32 vectors per chord. In the current experiments, 4,600 vector maps were recorded, and out of this dataset 650 vector maps contained features of the near wake behind the starling’s wing. The PIV data were measured 4 wing chord lengths (∼0.24 m) behind the right wing, so it took 20 ms for events generated at the wing to enter the PIV field of view. The wake was sampled in the parasagittal plane (9×9 cm^2^) at 2 ms intervals (500 Hz), so that both the downstroke and upstroke phases were temporally resolved.

**Figure 2:**
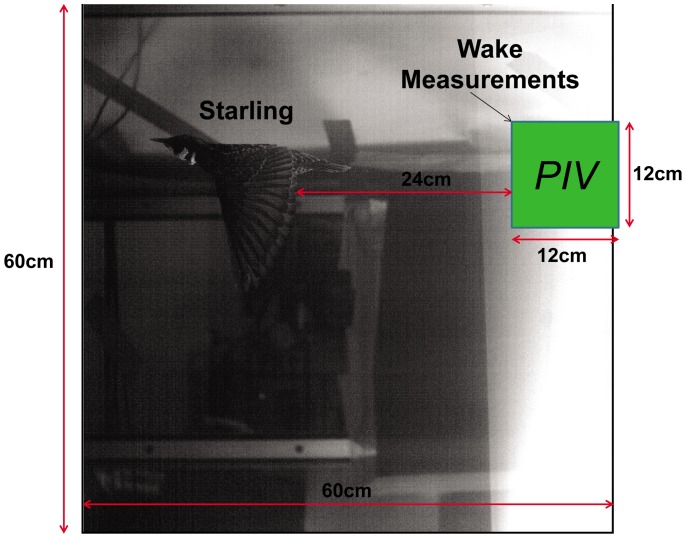
The large image shows the kinematic camera field of view and the small window marked “PIV” is the PIV camera field of view.

The streamwise and vertical positions of the bird were recorded simultaneously with the flow field measurements. The field of view in these recordings had an area of 9*c* by 9*c*. Figure 2 depicts a sample image of the starling flying in the tunnel as captured by the camera. The box marked with “PIV” indicates the location of the measured velocity fields from the PIV system. In addition, a floor-mounted camera operating at 60 Hz was used to record the spanwise position of the bird as well as the laser sheet illumination. These images allowed for the identification of the measured PIV plane in respect to the position of the wing; therefore, the wake velocity field could be associated with the spanwise location across the wing or the body. The floor-mounted camera was not synchronized with the PIV system, so the two time histories were synchronized manually based on the presence of laser light in the images. Once synchronized, spanwise positions were assigned to the wake data captured at 500 Hz based on interpolation from the simultaneously recorded spanwise positions recorded at 60 Hz.

An error analysis based on the root sum of squares method has been applied to the velocity data and the wing kinematics. The errors were estimated as: 2.5% for the instantaneous velocity values, 12% for the instantaneous vorticity and 3% for the drag values [40]. The error introduced in the kinematic analysis resulted from the spatial resolution of the image and the lens distortion leading to an estimated error of 5% in the wing displacements.

## Results

**Figure 3:**
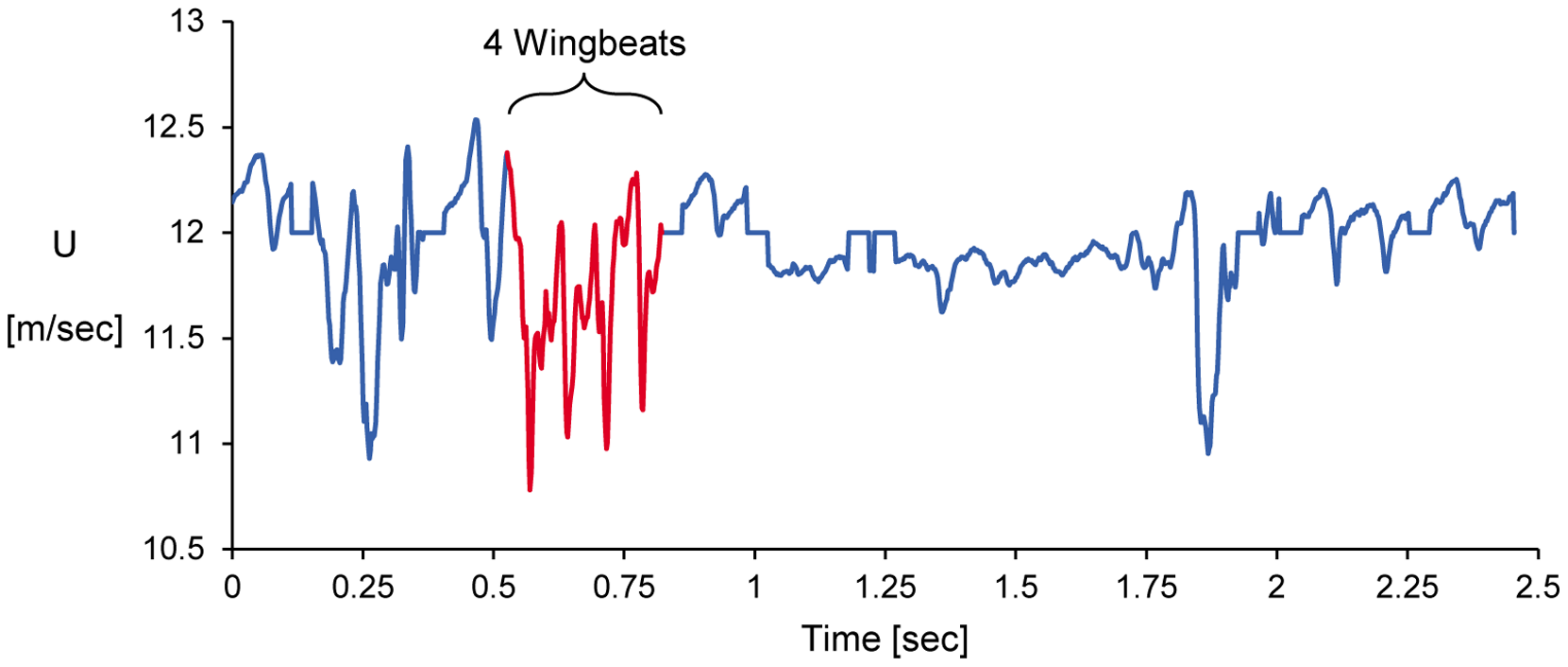
Variation of the average streamwise velocity in the wake with time.

A number of wake velocity maps were sampled where the starling was flying in a steady flapping mode without performing any maneuvers. The data discussed herein was selected from a broad acquisition batch where the bird was flying continuously for a few seconds (see Figure 3, where the streamwise velocity at the wake is depicted as a time series). The selection criterion was based on the flight mode chosen: no net acceleration of the bird over a wingbeat cycle as observed from the high speed imaging. The wing kinematics and the flow analysis are presented in the following sections. The analysis includes four sets of wingbeats each comprising a downstroke phase and an upstroke phase. The first three wingbeat sets contain a total of 110 velocity maps and kinematic images acquired simultaneously. These sets feature three consecutive wingbeats (referred to as wingbeats 1, 2 and 3, according to their order of appearance). A fourth wingbeat set (wingbeat 4) contains 43 vector maps and kinematic images.

### Kinematic Analysis

**Figure 4:**
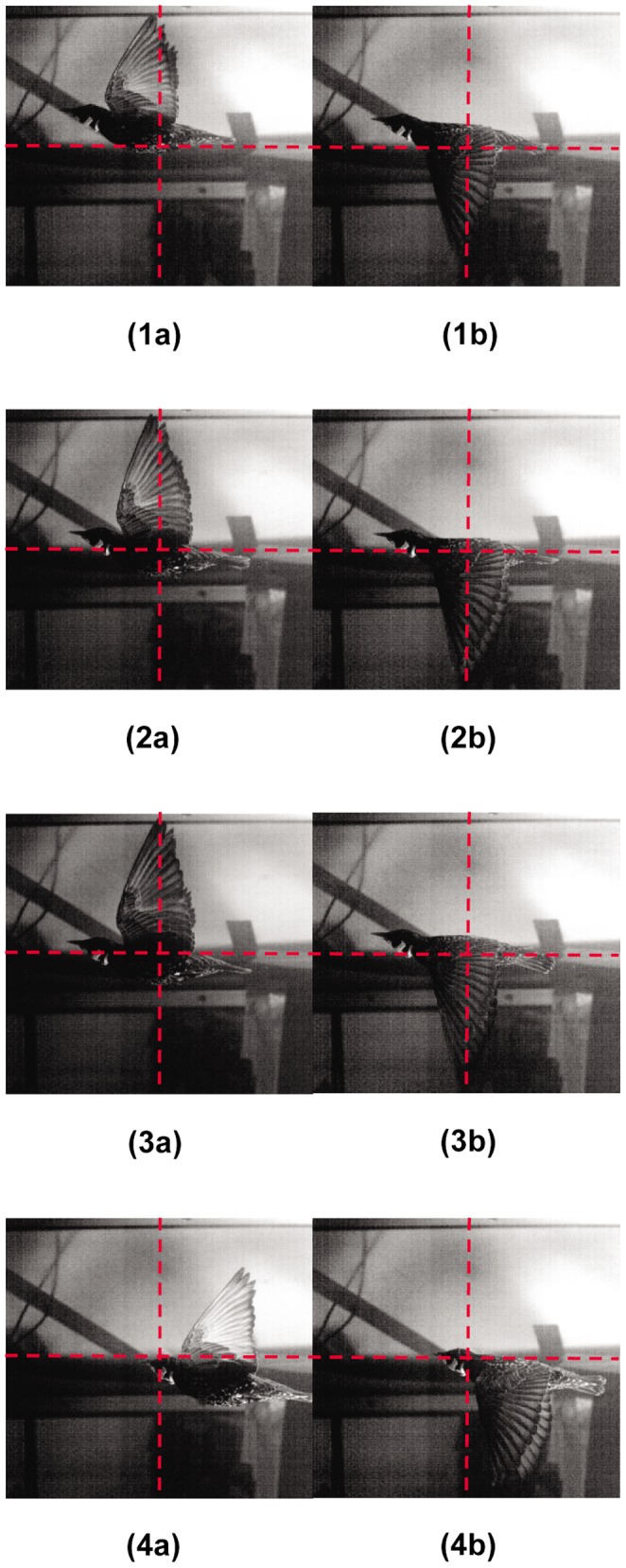
Different positions of the starling during the four wingbeats. The labels of the photographs correspond to the wingbeat number; ‘a’ marks the beginning of the downstroke, and ‘b’ marks the beginning of the upstroke. Each image center embedded with a red dashed cross lines for a visualization of the starling’s position during each wingbeat.

Figure 4 illustrates the starling in different positions during wingbeats 1, 2, 3 and 4. Since the purpose of this study is to estimate the streamwise forces acting on the bird, it is imperative that the bird accelerates negligibly in this direction. From the bird images shown in Figure 4, demonstrating different positions of the starling during the four wingbeats it is observed that, during each one of its four wingbeats, the starling does not accelerate noticeably in the streamwise direction. This is estimated by measuring the distance the bird travelled during the wingbeat cycle in the streamwise direction. The maximum displacement obtained for a complete wingbeat cycle is 35 mm, corresponding to wingbeat number 4. The other wingbeats displayed smaller displacements (around 10 mm). It should be noted that the observed starling motion was performed in a constant speed over the wingbeat cycles; i.e., there were no noticeable accelerations in the streamwise direction. For wingbeat 2, we have observed variations in the instantaneous velocity, yet the change in the bird displacement was less than 3 mm in the streamwise direction. For the maximum displacement of the starling the instantaneous velocity of the bird was 0.35 m/sec, which is significantly smaller, compared to the free stream velocity at the wing tunnel (12 m/sec). Therefore, such effects have negligible contribution to the results presented in this study.

Thus, analysis of the wake aerodynamics during these wingbeats can be performed assuming negligible acceleration of the bird in the streamwise direction.

In order to characterize the kinematics of the bird, several parameters have been estimated which are commonly used to evaluate bird flight characteristics beginning with the flapping frequency and Strouhal number [2] (see Table 1).

**Table 1:** The wingbeat frequency, Strouhal number and the wingtip angle of attack for the four wingbeat cycles.

Parameter	Wingbeat				Average*
	1	2	3	4	
*f* [*Hz*]	12.8	14.7	13.9	11.9	13.3
St	0.27	0.34	0.33	0.24	0.30
*φ* [*^o^*]	29	34	33	26	31

* The average value of each parameter for the all wingbeats.

Taylor et al. [41] suggest that, for a wide variety of animals (including fish, birds and insects), efficient cruising locomotion corresponds to a Strouhal number ranging between 0.2< St <0.4, and for the starling in cruising flight mode it should be about 0.3. The estimated Strouhal number averaged over four wing beat cycles ( = 0.3) as shown in Table 1 is in good agreement with Taylor et al. [41].

The wingtip angle of attack, *φ*, is computed by assuming that the wing moves up and down through its amplitude at a constant vertical speed
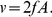
(1)


For a wing moving forward with a streamwise velocity *U*_∞_, the wingtip will move either up or down with a maximum ‘zigzag’ angle given by [42]:
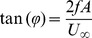
(2)


Substituting the Strouhal number in Eq. (2) yields:
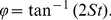
(3)


**Figure 5:**
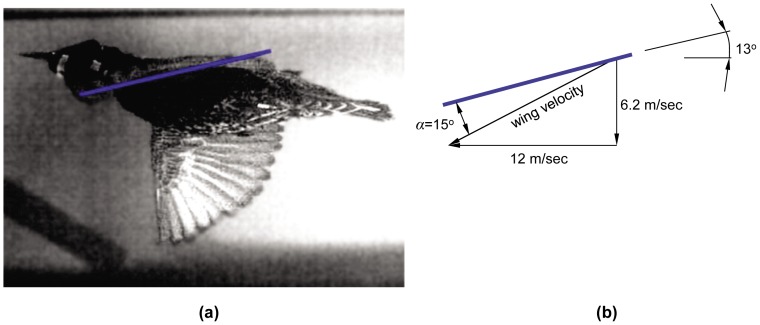
An estimation of the wing’s maximum angle of attack from its instantaneous pitch and velocity relative to the air. In (a), the left wing of the bird points directly at the camera, and the pitch of the chord line at approximately (2/3)*b*_semi_ is represented by the blue line. In (b), the pitch of the chord line and the velocity of the wing section at (2/3)*b*_semi_ are used to estimate the angle of attack, *α*, as approximately 15°.

For a bird flapping its wings up and down in the vertical plane and keeping the wing chord horizontal all the time (e.g., Figure 4), the wingtip angle of attack can be approximated as *φ* on the downstroke and - *φ* on the upstroke [42]. It is observed that the minimum mean wingtip angle of attack during the downstroke phase (*φ* = 26°) occurs for wingbeat 4 and that the value is larger than the stalling angle of conventional fixed wing aircraft of approximately 15° [43]. However, as shown by Nachtigall and Wieser [44], the angle of attack varies from zero at the shoulder to a maximum value at the wingtip. An additional assessment to determine the approximate angle of attack is performed based on the downstroke of the wing, as depicted in Figure 5. In Figure 5 the wing points directly towards the camera just after the tip has passed below the elevation of the shoulder. From the image, it is possible to estimate the pitch of the wing surface at approximately (2/3)*b*_semi_ where the chord length is greatest. This enables us to use the pitch of the wing (∼13°) and the wing’s tangential velocity to approximate the angle of attack, *α*. The instantaneous angle of attack is found to be approximately 15°.

### Wake Characteristics

**Figure 6:**
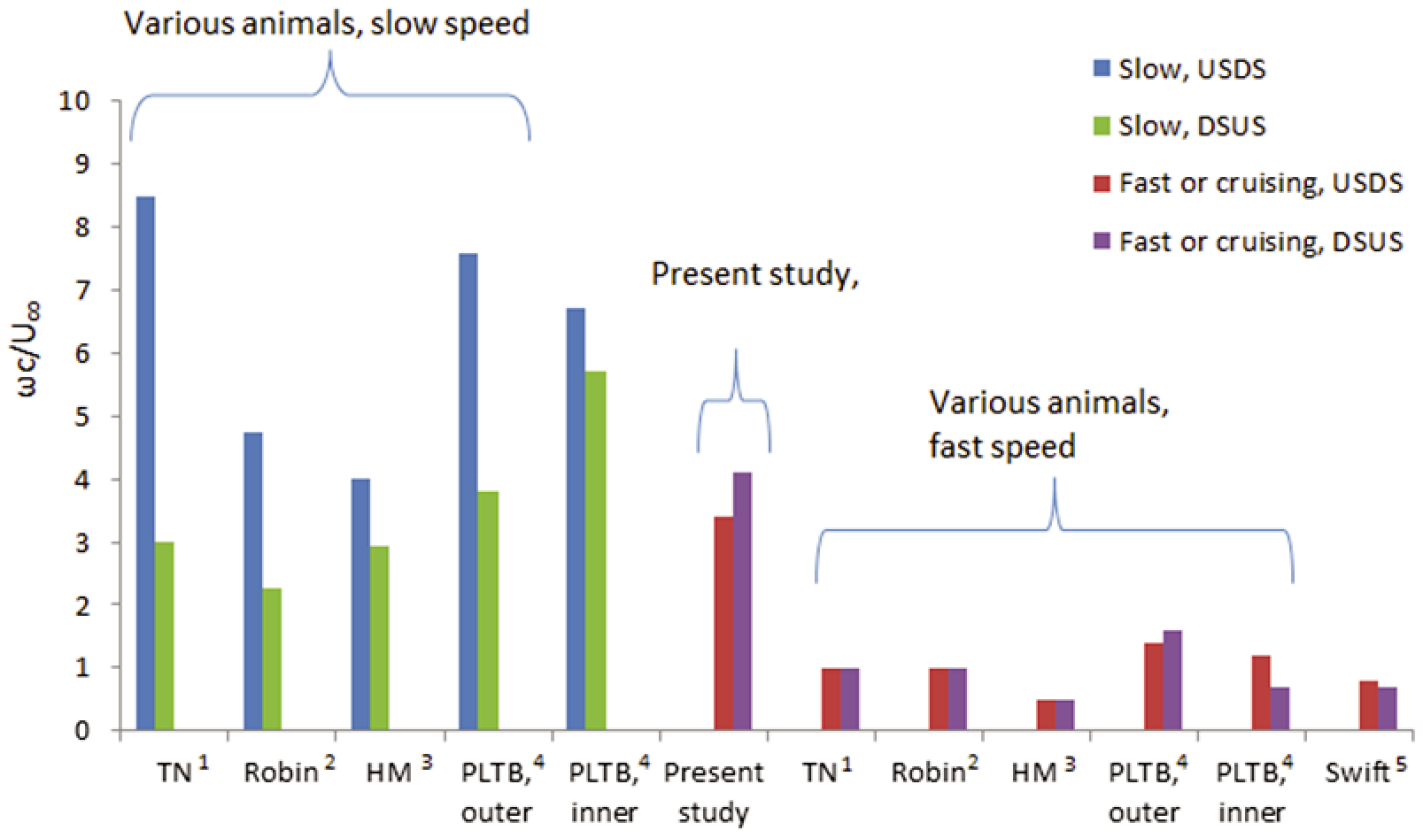
A comparison of the peak normalized spanwise vorticity, *ωc*/*U*_∞_, measured in the wake of flapping animals from several studies. Superscripts refer to the work of: 1 [20], 2 [22], 3 [45], 4 [4] and 5 [9]. Abbreviations used in the figure represent the Downstroke to upstroke transition (DSUS), upstroke to downstroke transition (USDS), the thrush nightingale (TN), house-martin (HM), and Pallas’ long-tongued bat (PLTB). Measurements from the Pallas’ long-tongued bat have been taken from the inner wing (*z*/*b*_semi_<0.4) and outer wing (*z*/*b*_semi_>0.75).

In the previous section the wing kinematics were quantified for each of the four complete wingbeats. In this section the wake is characterized in terms of aerodynamic forces and vorticity content. The vorticity in the wake is computed directly from the PIV data using a least squares differentiation scheme [40]. To determine if the vorticity as measured in the near wake is sufficient for the force estimations, the peak vorticity in the current data behind the starling are compared with that from previous works [4], [9], [20], [22], [45], as depicted in Figure 6. The peak spanwise vorticity measured in the wakes of several flapping wing animals is displayed in Figure 6 for the purpose of contextualizing the current measurements in the starling wake among other flapping wing animal studies. In Figure 6, the spanwise vorticity is normalized by the mean chord and wind speed of each respective study. Since peak vorticity measured in the wake of a cruising animal varies gradually over the range of flight speeds [20], peak values of spanwise vorticity are included for both extremes of the natural speed range where possible.

It is observed that the peak normalized vorticity of the starling is 4.1 in the present study (Figure 6), which is comparable with peak vorticity from birds and bats flying at the lower end of their natural speed range (blue and green bars). The values obtained herein are somewhat higher than values of peak vorticity from animals in cruising or fast flight (red and purple bars). A possible explanation for this difference is that the vorticity was measured at the near wake region in the current study while in many other birds studies the wake data were sampled farther downstream [20], and higher vorticity values are expected closer to the wake origin [46].

#### Wake reconstruction

During flapping flight bird wings change position causing the momentum and circulation in the wake to vary. In many simplified models the wake changes in a periodic manner where the downstroke and upstroke phases have different signatures [14], [20], [47]. In order to characterize the effect of the flapping action on the near wake behind the starling, and its impact on the aerodynamic performance, sequences of velocity maps have been reconstructed. This procedure was performed using PIV data collected at a sampling rate of 500 Hz – significantly higher than the 13.3 Hz wingbeat frequency. Therefore, a pattern of vorticity appearing in one frame also appears in the consecutive frame – only phase-shifted. The wake composite is formed by plotting sequential instantaneous vorticity fields computed from PIV data and by matching shifted patterns in the vorticity fields. The offset of the n^th^ successive PIV images is calculated as U_c_⋅Δt⋅n. The convection velocity, U_c_, is the velocity at which the characteristics of the wake collectively travel downstream. In the present study, wake composites have been generated using the free-stream velocity (U_∞_) as a convection velocity. The generation of a wake composite provides a useful visualization tool for observation of the wake dynamics over the time series of a wing beat cycle. What appears as “downstream” in the wake composite happens earlier, while what appears “upstream” in the composite happens later meaning that the generation of the wake composite invokes Taylor’s hypothesis in which the characteristics of the flow are a frozen spatial pattern convected through the field of view.

**Figure 7:**
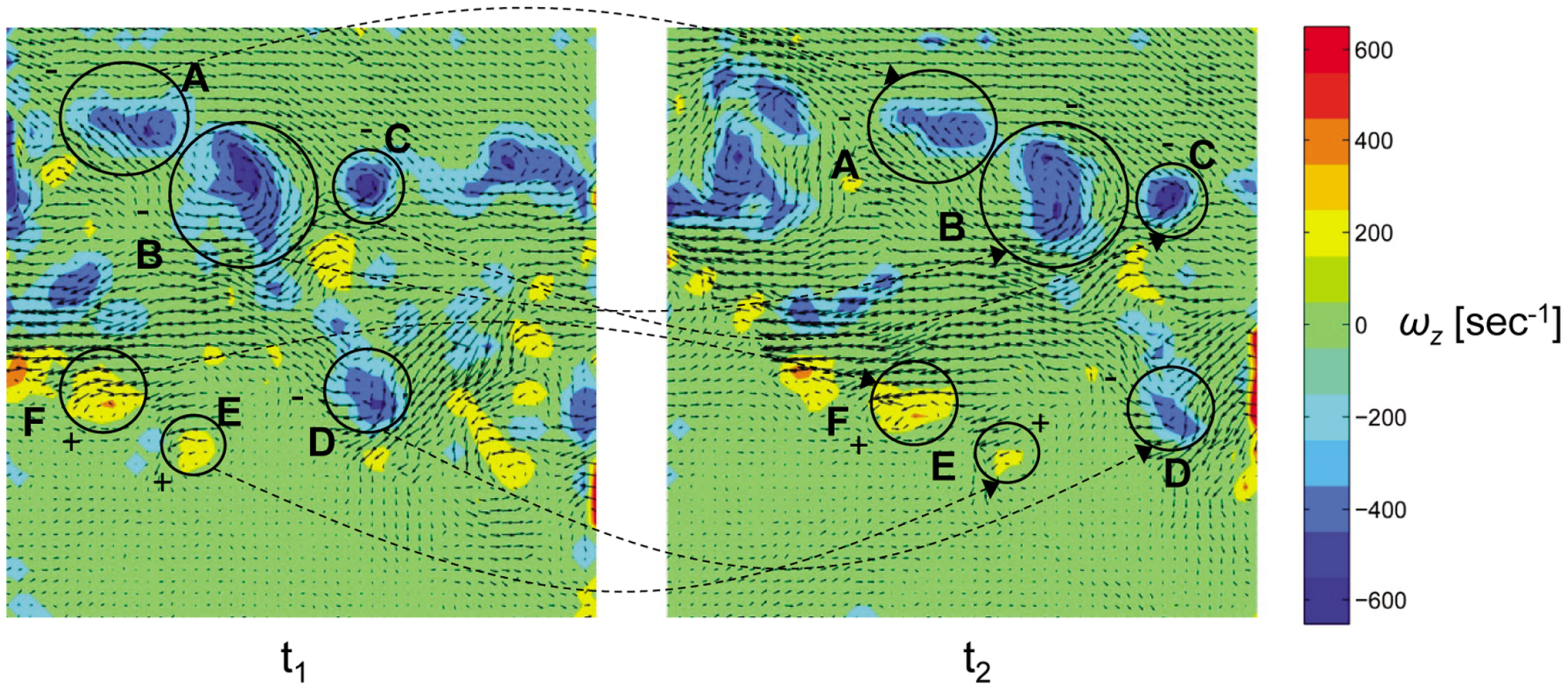
Two consecutive spanwise vorticity fields (t_2_ = t_1_+2 msec). The air flows from left to right and each frame size is 9×9 cm^2^.

Figure 7 demonstrates the wake reconstruction procedure: initially two consecutive spanwise vorticity fields, along with the velocity fluctuations, are put side by side. Afterwards, a vorticity pattern classification process is performed on each image, which eventually assists in the identification of similar patterns between the two images according to similarity in size, shape, direction and value. Figure 7 shows four different negatively-signed (*A*–*D*) patterns and two different positively-signed (*E* and *F*) patterns. It can be seen that the different patterns of vorticity move downstream during the time difference between the consecutive images (2 msec).

#### Wake evolution

**Figure 8:**
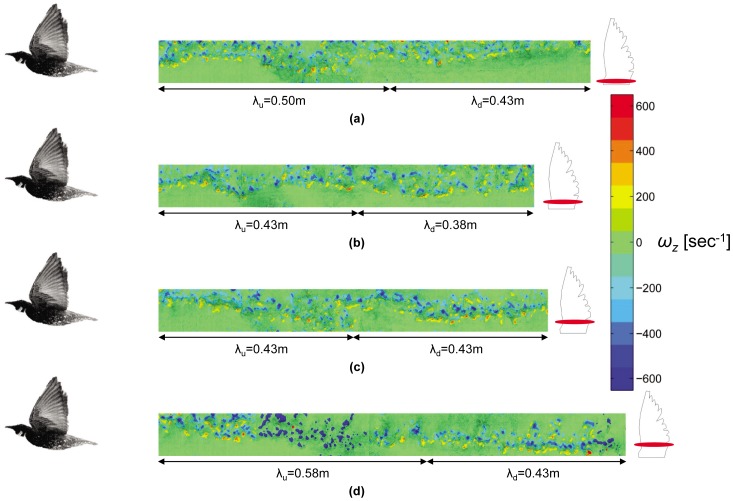
Reconstruction of the starling’s wake consisting of four wingbeats as though the starling flew from right to left. The average spatial flow has been subtracted, thus the vectors displayed are the velocity fluctuations. The contours represent the spanwise vorticity in each wingbeat and the half-wavelengths for the downstroke (λ_d_) and the upstroke (λ_u_) are noted for each wingbeat. Wingbeat numbers go from top to bottom: (a) 1, (b) 2, (c) 3, and (d) 4.

The wake features are presented through fluctuating velocity and vorticity fields as depicted in Figure 8. The set of figures describes the wake of the freely flying starling during the four different wingbeats (as defined in the kinematic analysis chapter). Each wake pattern consists of 24 consecutive fields displaying the spanwise vorticity, *ω_z_*(*x*, *y*), varying from −650 to 650 sec^−1^. In addition, the spatially averaged velocity has been subtracted from each frame, so the velocity vectors displayed are fluctuations.

**Figure 9:**
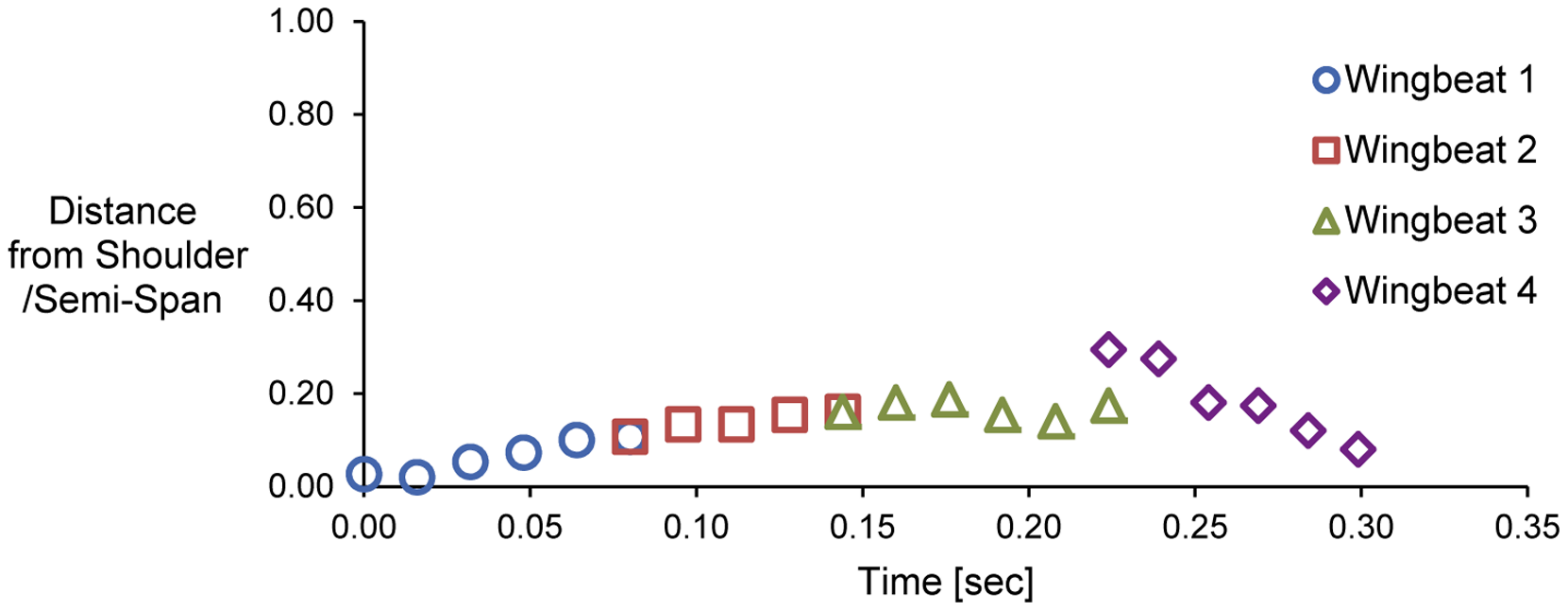
The location of the laser sheet with respect to the right wing during wingbeats 1, 2, 3 and 4.

Using the floor-mounted camera, the wake patterns presented in Figure 8 were determined to be captured in a plane that is, on average, approximately 2.5 cm from the right wing root (14% of the wing length). Figure 9 demonstrates the different wing sections being intersected with the laser sheet as a consequence of the starling’s small spanwise movements throughout the wingbeats. It should be noted that, in the four wingbeats presented in this study, the starling was recorded with the minimum possible spanwise, vertical and streamwise movements.

The most immediate observation from the wake reconstructions in Figure 8 is the periodicity of the wake over the shedding cycle. In light of the topology of spanwise vorticity, it is convenient to discuss the wake in terms of a top half and a bottom half where the wake center is defined by the location of largest velocity deficit. As would be expected, the top half of the wake is composed primarily of negative spanwise vorticity and the bottom half of the wake by positive spanwise vorticity. It is also noted that the quantity of lift producing vorticity (i.e., negative) is greater than that of positive vorticity.

In the available literature, there has been considerable focus on the vortex topology in bird wakes [20], [22], [30]. A comparison with these works shows that there are qualitative similarities in the vorticity structure between the current measurements close to the body (along the span) and those shown by Henningsson et al. [9] for a swift. From their measurements of the wake at 10 chord lengths downstream of a flying swift, Henningsson et al. [9] suggest that the tip vortices for the swift are connected by spanwise vortices. The measurement plane in the current study is significantly closer to the bird in the streamwise direction (4 chord lengths) offering a new perspective on wake development. Distinguishing coherent vortices in complex flows is not trivial, and this topic has been investigated extensively [47]–[49]. Bird wakes, like all wakes, are examples of free shear flow [50], where vorticity occurs in the wake due to the formation of a strong velocity gradient (i.e., *∂u/∂y*). However, it is known that vorticity alone does not indicate the presence of a vortex [47]. In the current study, the resolution of the measurements makes it difficult to determine whether the computed vorticity is associated with coherent structures or high shear. Therefore, if there are spanwise connecting vortices at this early stage of wake development, they must be smaller than the smallest resolvable vortices based on the given PIV resolution, which for the current measurements means they must be smaller than 4 mm. In the next section, the wake data are used to quantitatively examine the sectional drag force without relying on vortex-based methods.

### Drag Estimates

**Figure 10:**
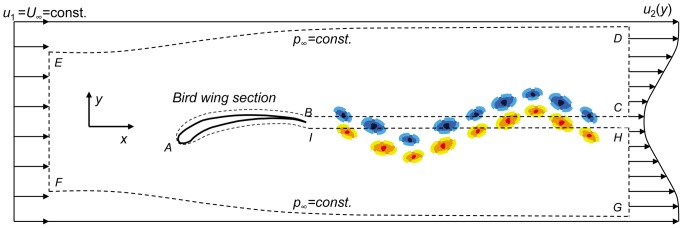
Control volume around a two-dimensional bird wing section in a uniform free stream.

Consider a two-dimensional section of the bird wing in an incompressible flow as sketched in Figure 10. The wing is located within a control volume (*ABCDEFGHIA*) with its width in the *z* direction being unity. Inside the control volume, the integral form of the momentum equation is [51]

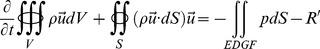
(4)where 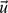 is the velocity, *ρ* is the density (constant), *p* is the pressure and *R′* is the resultant aerodynamic force per unit span exerted on the body by the normal and shear stresses acting at the body surface. Note that the viscous terms have been ignored as they scale with Re^−1^. The integrals are taken over the control volume, *V*, enclosed by the surface, *S*. Using the *x*-component of Eq. (4), the aerodynamic drag per unit span, *D′*, is
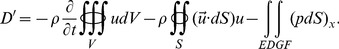
(5)


A positive value of *D′* is defined as drag and a negative one as thrust. For simplicity the resultant force, *D′*, is referred to as drag per unit span where negative drag per unit span refers to thrust. For *S*, estimated sufficiently far from the body where the pressure is assumed constant, and equal to the undisturbed free-stream pressure *p*_∞_, Eq. (5) becomes:
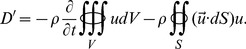
(6)


By definition, 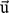 is parallel to the streamlines and *dS* is perpendicular to the control surface. Thus, for streamlines *ED*, *FG* and *BAI* the multiplication 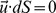. In addition, the planes *BC* and *HI* are adjacent to each other, so their contribution to the second term in Eq. (6) cancels each other. As a result, the second term in Eq. (6) consists of contributions only from sections *EF* and *DG* (where 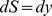), and becomes
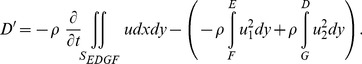
(7)


Using the integral form of the continuity equation and multiplying by *u*_1_ (a constant in the current case),
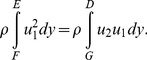
(8)and substituting Eq. (8) into Eq. (7) leads to



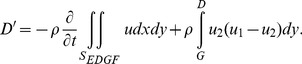
(9)Therefore, the drag is composed of two terms: steady (second term) and unsteady (first term). The steady drag per unit span, referred to as the velocity deficit drag in classical aerodynamics, can be derived from the second term in Eq. (9) and expressed as
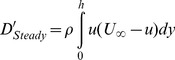
(10)where *h* is the wake vertical extent of the PIV velocity field. The so-called ‘unsteady drag’ per unit span can be derived using the first term in Eq. (9) and expressed as
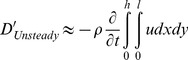
(11)where l is the streamwise extent of the PIV velocity field.

**Figure 11:**
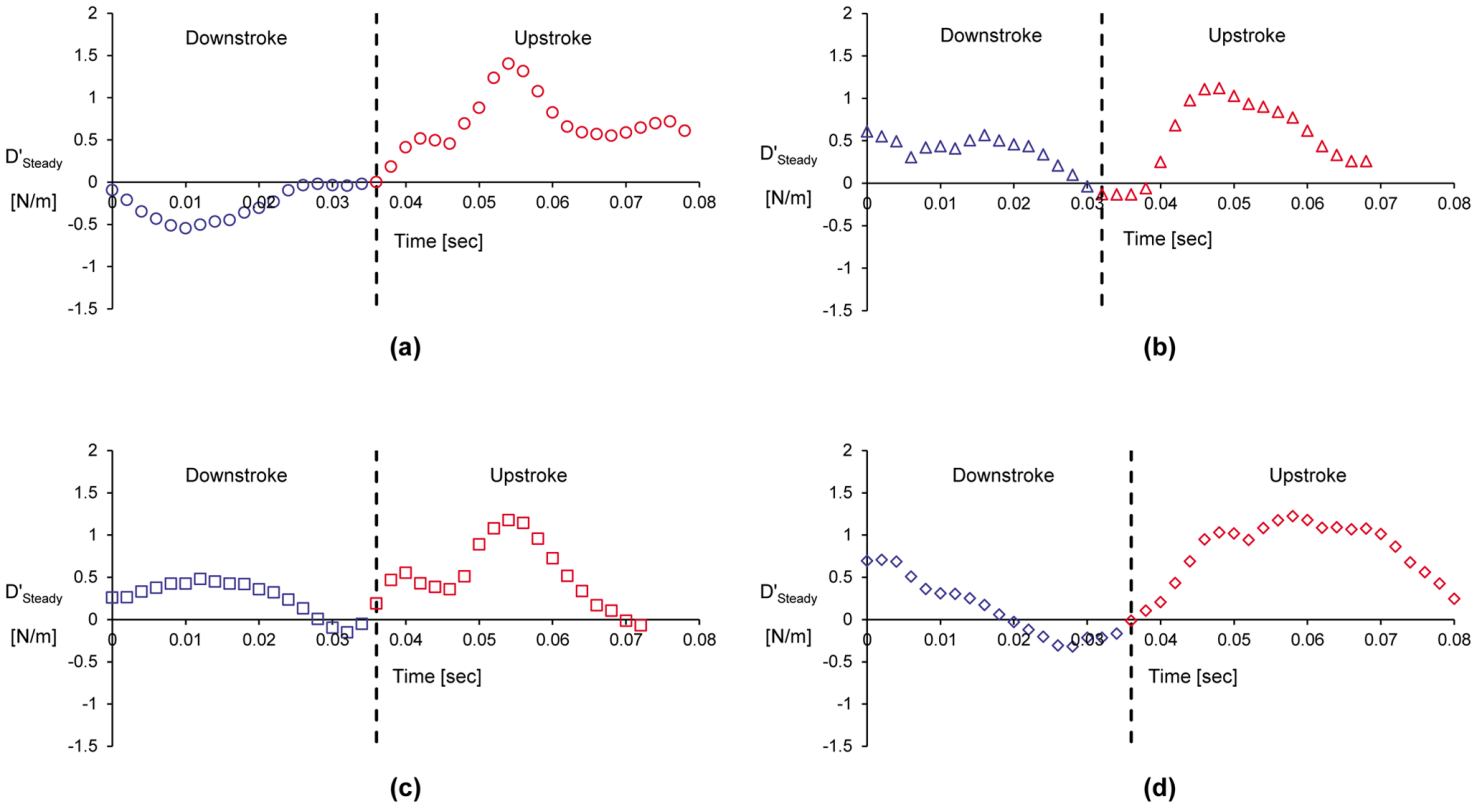
Steady drag per unit span versus time, as computed according to Eq. (10), for wingbeats no. 1 (a), 2 (b), 3 (c) and 4 (d).

**Figure 12:**
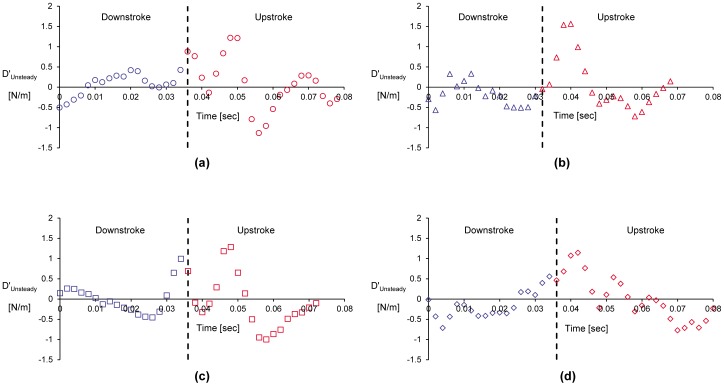
Unsteady drag per unit span versus time, as computed according to Eq. (11), for wingbeats no. 1 (a), 2 (b), 3 (c) and 4 (d).

**Figure 13:**
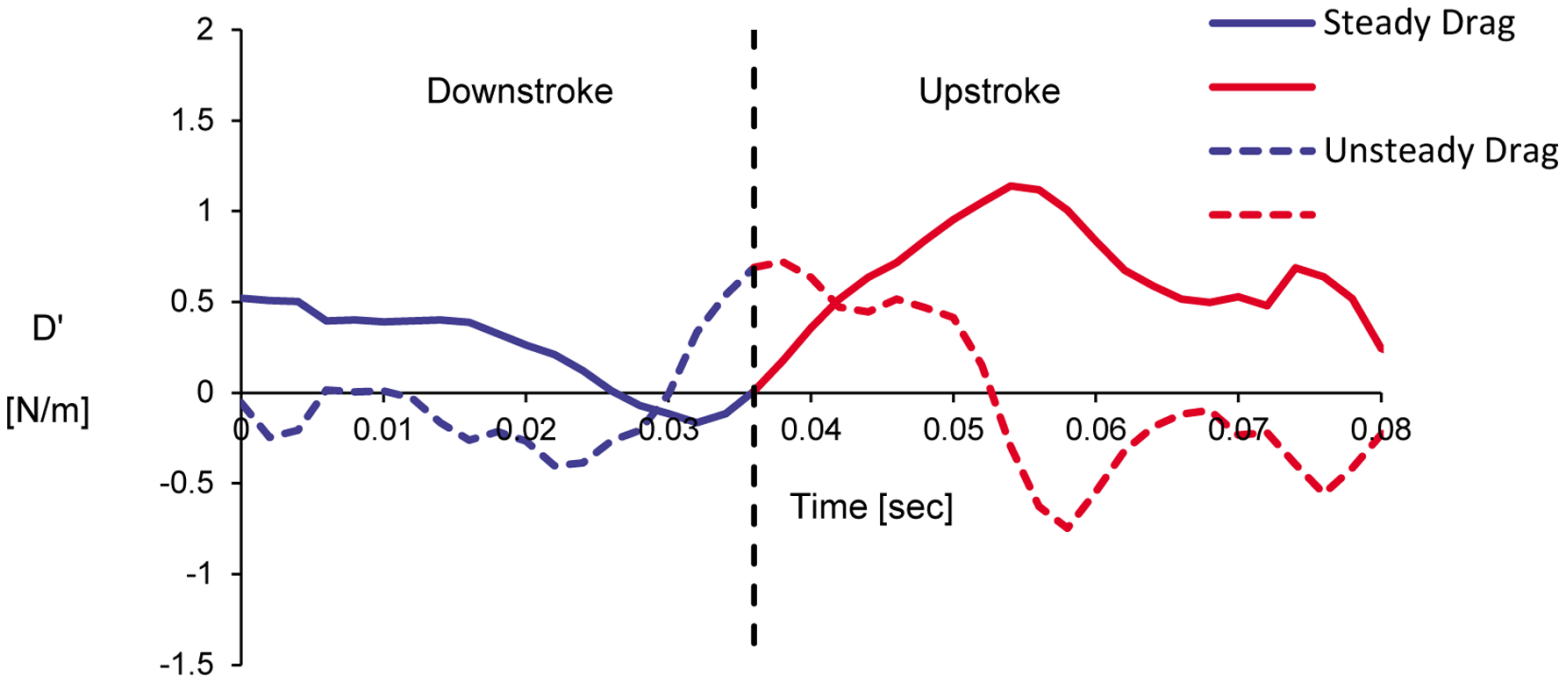
Averaged steady and unsteady drag per unit span versus time, as computed according to Eq. (10) and (11).

It should be noted that the full area integral (or, volume integral per unit span) cannot be directly computed as it appears in Eq. (9) since only a portion of the control surface enveloping the bird wing is measured in the current experiments (e.g., Figure 2). However, Eq. (11) is used as an approximation to the entire area integral bound by the control surface shown in Figure 10. Figures 11 and 12 describe the time variation of the steady and unsteady drag per unit span as computed from Eq. (10) and (11) for the four different wingbeats (see a–d in Figures 11 and 12) as depicted in Figure 8. The integrals were performed for each instantaneous velocity field of the starling’s wake. For the steady drag per unit span, different streamwise velocity profiles were sampled at different *x*-positions for each velocity field map. Subsequently, these profiles within one PIV vector map were spatially averaged into one profile describing the velocity deficit (steady drag per unit span) similar to the procedure described in [18]. This procedure is similar to a spatial windowing average in order to smooth out some of the variations within each vector map, and it is noted that the general trend over the wingbeat cycles does not change using this procedure. Each point in Figures 11 and 12 represents the integral value depicted from each velocity field yielding a time evolution of the drag in the near wake. Figure 13 depicts the averaged steady and unsteady drag profiles for the four wingbeats. The uncertainty of the computed drag values in Figures 11 and 12, estimated in experimental setup section, is similar to the size of the markers. Notable differences are observed between the steady and unsteady components of the horizontal momentum in Figures 11 and 12. Figure 13 shows that the averaged steady drag is positive over the entire wingbeat cycle except for a short period in the transition from downstroke to upstroke. Contrary to the steady portion, the unsteady contribution to the drag, as depicted in Figure 13, is negative during both the downstroke and upstroke while positive during the transition phase. The steady drag values reach 1 and 0.5 N/m during the upstroke and downstroke, respectively. The negative values as calculated for the unsteady portion reach a minimum value of −0.5 N/m. These differences are discussed in detail in the following section.

## Discussion

In classical aerodynamics, the profile drag (Eq. (10)) inherently assumes that *∂u/∂t = *0 everywhere in the chosen control volume. While this assumption is reasonable in the wake of a section model mounted in a wind tunnel, it seems unlikely that this condition is satisfied in the wake of a freely flying bird. However, experimental measurements of the entire control volume remain prohibitive and many studies have approximated the drag force through the profile drag [20], [50]. In the current study, the profile drag has been estimated in the same manner over four complete wingbeats of a freely flying starling (Figure 11). The steady drag was demonstrated to vary significantly during the wingbeat cycle yielding higher profile drag during the upstroke. This variation in the drag force over the wingbeat cycle is consistent with earlier studies [52], which suggests that the upstroke phase generates more drag than the downstroke phase.

**Figure 14:**
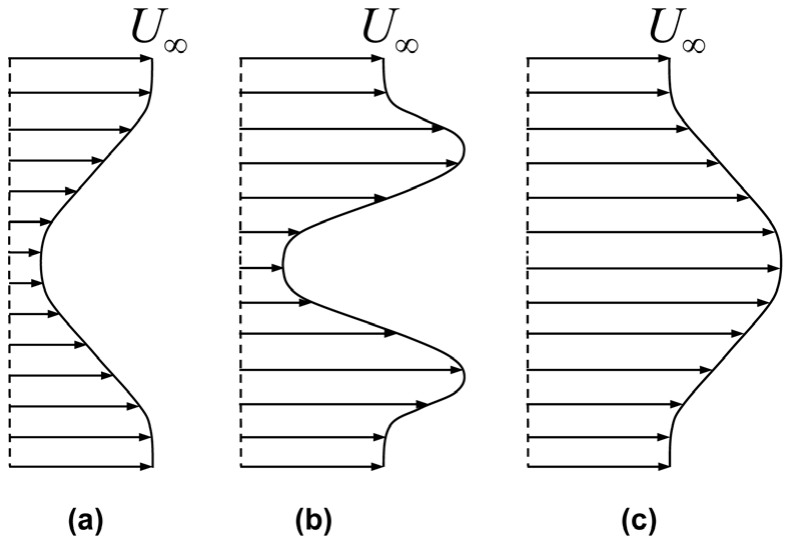
Schematic examples of a drag wake (a), momentumless wake (b) and a jet wake (c).

The profile drag developed over the wing is manifested through the wake velocity profile. In the case of flapping wings (or in any event where the flow is disturbed by some external motion), the velocity field changes spatially and temporally. Since the bird is flying freely, and the kinematic images (Figure 4) demonstrate that the bird does not accelerate in the streamwise direction, there is no net momentum change in the streamwise direction. Since the profile drag is the term relevant to the overall streamwise momentum balance of Eq. (4) then one would expect a momentumless velocity profile (see Figure 14). However, as indicated by the profile drag, which is generally positive (Figure 13), as well as in profiles presented in previous studies [20], it appears that another force is required to balance the streamwise momentum.

From the momentum balance (Eq. (4)) it is expected that the compensating force for the profile drag is the volume integral containing the *∂u/∂t* term. Considering the control volume drawn in Figure 10, it is plausible to assume that *∂u/∂t* = 0 everywhere in the flow upstream of the wing since the wind tunnel is operating at a constant wind speed. Conversely, *∂u/∂t* is not expected to be zero around the wings and especially in the wake. It is expected that *∂u/∂t* is greater than zero in the wake during the downstroke since work (e.g., the flapping motion) is an input to the wake [51]. However, during the upstroke it remains unclear if this term is negative or positive. The estimate of the ‘unsteady drag’ (Eq. (11); Figure 12) uses a portion of the volume as an approximation to the total volume integral of *∂u/∂t.* The unsteady drag results presented in Figure 13 demonstrate that *∂u/∂t* is smaller than zero as expected during the downstroke as the bird generates lift and propels itself forward [51]. At the beginning of the upstroke, the volume integral of *∂u/∂t* appears to be positive; however, once the steady drag reach a maximum value halfway through the upstroke (Figure 13), the unsteady drag once more becomes negative indicating that *∂u/∂t* >0, as it was during the downstroke.

It should be noted that performing PIV measurements around freely flying birds limits the capability of capturing the entire span of the wing; therefore, our estimates are based on a sectional measurement. However, the current dataset offers much lower uncertainty of the streamwise velocity compared to attempts to capture the entire volume using the Trefftz plane [27]. Drag variations may also arise due to the wing flexing, which acts to minimize the drag during the second half of the upstroke phase [2]. In addition, it is well known that a significant amount of thrust is generated at the outer part of the wing [2]. However, the time-resolved streamwise velocity measurements in the near wake of a freely flying starling suggest that for a complete understanding of the drag and thrust relationship in bird wakes the importance of *∂u/∂t* cannot be neglected. Furthermore, the flow mechanisms underlying the present measurements of *∂u/∂t* are expected to correlate to the bird’s use of unsteady aerodynamics and improved efficiency.

Many studies that investigate flying animals utilize quasi-steady models to analyze the flapping mechanism [18], [20], [51]. Quasi-steady analysis of flapping flight generally assumes that the aerodynamic forces in flapping flight can be composed from the various instantaneous wing configurations, as they would behave in an equivalent series of steady flows. However, our estimation of the steady drag force shows that it is mostly positive indicating that the bird should be decelerating if acted on by this force alone. The kinematic observations show that the bird is not accelerating in the streamwise direction, so there should be a balancing force that is not accounted for in the steady aerodynamics. The approximation of the unsteady contribution to the streamwise force indicates that the balancing force to the steady velocity deficit drag is most likely due to unsteady aerodynamics, thus revealing an inadequacy of quasi-steady approaches. Future studies measuring a greater spanwise distribution of drag forces are necessary to obtain the complete description of steady versus unsteady aerodynamics.

## Conclusions

In this study, a long-duration time-resolved PIV system was used to obtain accurate, time-resolved measurements of the streamwise velocity in the wake of a freely flying European starling flying in flapping flight at the AFAR hypobaric wind tunnel. The system is capable of capturing images for 20 min continuously in order to characterize unsteady phenomena within a given flow field. A total of 4,600 vector maps were analyzed in the current study, and four wingbeat cycles were identified within this data set. The identification was performed by using an additional high-speed camera that recorded flight kinematics and was synchronized with the PIV. The kinematic analysis showed that during the four wingbeats used to analyze the wake, the bird did not accelerate or decelerate significantly in the streamwise direction.

The wake topology of the starling was characterized using a wake reconstruction based on patterns from the instantaneous vorticity fields where the measurement plane of the velocity was close to the wing root of the bird. The resolution of the data were insufficient to determine if spanwise vortices were present in the near wake or if the observed vorticity was due to the shear created by the flow over the bird’s wings and body. Thus, any connecting spanwise vortices that exist at this stage of the wake development should be relatively small.

The time variation of the profile drag per unit span from classical aerodynamics over each of the four different wingbeat cycles was approximated using the PIV data. Inherent in the calculation of profile drag is the assumption that *∂u/∂t* is zero everywhere or integrates to zero instantaneously, yet this does not necessarily correspond to the case for flapping flight. As with previous studies, the integration of the velocity profiles over the measurement plane admittedly misses the remaining span of the wake, yet clear trends have been observed. It was found that the profile drag term was almost always positive; however, the bird was not observed to noticeably decelerate. Thus, there should be a compensating force to this classical drag term. It was observed that *∂u/∂t* is generally negative during the downstroke from the current dataset; however, the results also show that during the upstroke *∂u/∂t* is generally positive. The approximation of the unsteady term suggests that unsteady aerodynamics may provide some thrust in the overall streamwise force balance.

The role of the unsteady portion of the flow on the flight efficiency of birds is yet to be determined and still remains an open question. Yet, the current results shed light on the role of the unsteadiness during flight and its impact on drag/thrust. In addition, future studies are required to assess how the spanwise variations of these forces affect the balance between drag and thrust.

## References

[1] Zheng L, Hedrick T, Mittal R (2013) A comparative study of the hovering efficiency of flapping and revolving wings. Bioinspir Biomim 8: 036001. 23680659 10.1088/1748-3182/8/3/036001

[2] Shyy W, Lian Y, Tang J, Viieru D, Liu H (2008) Aerodynamics of low Reynolds number flyers. Cambridge University Press.

[3] Floreano D, Zufferey JC, Srinivasan MV, Ellington C (2009) Flying Insects and Robots. Springer.

[4] Hedenström A, Spedding GR (2008) Beyond robins: aerodynamic analyses of animal flight. J R Soc Interface 5: 595–601. 18397865 10.1098/rsif.2008.0027 PMC2706015

[5] Wagner H (1925) Über die entstehung des dynamischen auftriebes von tragflügeln. ZAMM 5(1): 17–35.

[6] Theodorsen T (1935) General theory of aerodynamic instability and the mechanism of flutter. NACA Tech Rep No 496.

[7] Brown RHJ (1963) The flight of birds. Biol Rev 38: 460–489.

[8] Brunton S, Rowley C (2010) Unsteady aerodynamic models for agile flight at low Reynolds numbers. 48th AIAA Aerospace Sciences Meeting and Exhibit.

[9] Henningsson P, Spedding GR, Hedenström A (2008) Vortex wake and flight kinematics of a swift in cruising flight in a wind tunnel. J Exp Biol 211: 717–730. 18281334 10.1242/jeb.012146

[10] Lehmann FO (2004) The mechanisms of lift enhancement in insect flight. Naturwissenschaften 91: 101–122. 15034660 10.1007/s00114-004-0502-3

[11] Muijres FT, Johansson LC, Barfield R, Wolf M, Spedding GR, et al (2008) Leading-edge vortex improves lift in slow-flying bats. Science 319: 1250–1253. 18309085 10.1126/science.1153019

[12] Hubel TY, Tropea C (2009) Experimental investigation of a flapping wing model. Exp Fluids 46: 945–961.

[13] Rayner JMV (1979) A vortex theory of animal flight. I. The vortex wake of a hovering animal. J Fluid Mech 91: 697–730.

[14] Rayner JMV (1979) A vortex theory of animal flight. II. The forward flight of birds. J Fluid Mech 91: 731–763.

[15] Rayner JMV (1979) A new approach to animal flight mechanics. J Exp Biol 80: 17–54.

[16] Spedding GR (1986) The wake of a jackdaw (*Corvus monedula*) in slow flight. J Exp Biol 125: 287–307.

[17] Spedding GR (1987) The wake of a kestrel (*Falco tinnunculus*) in flapping flight. J Exp Biol 127: 59–78.

[18] Rayner JMV (2001) Mathematical modeling of the avian flight power curve. Math Meth Appl Sci 24: 1485–1514.

[19] Hedrick TL, Tobalske BW, Biewener AA (2002) Estimates of circulation and gait change based on a three-dimensional kinematic analysis of flight in cockatiels (*Nymphicus hollandicus*) and ringed turtle-doves (*Streptopelia risoria*). J Exp Biol 205: 1389–1409. 11976351 10.1242/jeb.205.10.1389

[20] Spedding GR, Rosén M, Hedenström A (2003) A family of vortex wakes generated by a thrush nightingale in free flight in a wind tunnel over its entire natural range of flight speeds. J Exp Biol 206: 2313–2344. 12796450 10.1242/jeb.00423

[21] Rosén M, Spedding GR, Hedenström A (2004) The relationship between wingbeat kinematics and vortex wake of a thrush nightingale. J Exp Biol 207: 4255–4268. 15531647 10.1242/jeb.01283

[22] Hedenström A, Rosén M, Spedding GR (2006) Vortex wakes generated by robins *Erithacus rubecula* during free flight in a wind tunnel. J R Soc Interface 3: 263–276. 16849236 10.1098/rsif.2005.0091 PMC1578743

[23] Johansson LC, Wolf M, von Busse R, Winter Y, Spedding GR, et al (2008) The near and far wake of pallas' long tongued bat (*Glossophaga soricina*). J Exp Biol 211: 2909–2918. 18775928 10.1242/jeb.018192

[24] Rayner JMV, Viscardi PW, Ward S, Speakman RS (2001) Aerodynamics and energetics of intermittent flight in birds. Amer Zool 41: 188–204.

[25] Hedenström A, Muijres FT, von Busse R, Johansson LC, Winter Y, et al (2009) High-speed stereo DPIV measurement of wakes of two bat species flying freely in a wind tunnel. Exp Fluids 46: 923–932.

[26] Hubel TY, Hristov NI, Swartz SM, Breuer KS (2009) Time-resolved wake structure and kinematics of bat flight. Exp Fluids 46: 933–943.

[27] Waldman RM, Breuer KS (2012) Accurate measurement of streamwise vortices using dual-plane PIV. Exp Fluids 53: 1487–1500.

[28] Hubel TY, Riskin DK, Swartz SM, Breuer KS (2010) Wake structure and wing kinematics: the flight of the lesser dog-faced fruit bat, *Cynopterus brachyotis* . J Exp Biol 213: 3427–3440. 20889823 10.1242/jeb.043257

[29] Henningsson P, Muijres FT, Hedenström A (2010) Time-resolved vortex wake of a common swift flying over a range of flight speeds. J R Soc Interface 8: 807–816. 21131333 10.1098/rsif.2010.0533 PMC3104350

[30] Johansson LC, Hedenström A (2009) The vortex wake of blackcaps (*Sylvia atricapilla L.*) measured using high-speed digital particle image velocimetry (DPIV). J Exp Biol 212: 3365–3376. 19801441 10.1242/jeb.034454

[31] Muijres FT, Johansson LC, Winter Y, Hedenström A (2011) Comparative aerodynamic performance of flapping flight in two bat species using time-resolved wake visualization. J R Soc Interface 8: 1418–1428. 21367776 10.1098/rsif.2011.0015 PMC3163419

[32] Muijres FT, Spedding GR, Winter Y, Hedenström A (2011) Actuator disk model and span efficiency of flapping flight in bats based on time-resolved PIV measurements. Exp Fluids 51(2): 511–525.

[33] Sollof SM, Adrain RJ, Liu ZC (1997) Distortion compensation for generalized stereoscopic particle image velocimetry. Meas Sci and Tech 8: 1441–1454.

[34] van Doorne CWH, Westerweel J (2007) Measurement of laminar, transitional and turbulent pipe flow using Stereoscopic-PIV. Exp Fluids 42: 259–279.

[35] Hubel TY, Tropea C (2010) The importance of leading edge vortices under simplified flapping flight conditions at the size scale of birds. J Exp Biol 213: 1930–1939. 20472780 10.1242/jeb.040857

[36] Hall KC, Hall S (2001) A rational engineering analysis of the efficiency of flapping flight. Ed. by Mueller T.J., Fixed and flapping wing aerodynamics for micro air vehicle applications, AIAA, Ch. 13, 249–273.

[37] Kirchhefer A, Kopp GA, Gurka R (2013) The near wake of a freely flying European starling. Phys. Fluids 25: 051902. 10.1371/journal.pone.0080086 PMC3838395 24278243

[38] Taylor ZJ, Gurka R, Kopp GA, Liberzon A (2010) Long-duration time-resolved PIV to study unsteady aerodynamics. IEEE Transactions on Instrumentations and Measurement 59: 3262–3269.

[39] Echols WE, Young JA (1963) Studies of portable air-operated aerosol generators. NRL Tech Rep.

[40] Raffel M, Willert CE, Kompenhans J (1998) Particle image velocimetry: a practical guide. Springer.

[41] Taylor GK, Nudds RL, Thomas ALR (2003) Flying and swimming animals cruise at a Strouhal number tuned for high power efficiency. Nature 425: 707–711. 14562101 10.1038/nature02000

[42] Linton JO (2007) The physics of flight: II. Flapping wings. Physics Education 42: 358–364.

[43] Tennekes H (2009) The simple science of flight: from insects to jumbo jets. MIT Press, London.

[44] Nachtigall HVW, Wieser J (1966) Profilmessungen am Taubenflugel. Zeit Vergl Physiol 52: 333–346.

[45] Rosén M, Spedding GR, Hedenström A (2007) Wake structure and wingbeat kinematics of a house-martin Delichon urbica. J R Soc Interface 4: 659–668. 17264054 10.1098/rsif.2007.0215 PMC2373391

[46] Dong H, Mittal R, Najja FM (2006) Wake topology and hydrodynamic performance of low-aspect-ratio flapping foils. J Fluid Mech 566: 309–343.

[47] Vernet A, Kopp GA, Ferré JA, Giralt F (1999) Three-dimensional structure and momentum transfer in a turbulent cylinder wake. J Fluid Mech 394: 303–337.

[48] Jeong J, Hussain F (1995) On the identification of a vortex. J Fluid Mech 285: 69–94.

[49] Chakraborty P, Balachandar S, Adrian RJ (2005) On the relationships between local vortex identification schemes. J Fluid Mech 535: 189–214.

[50] Spedding GR, Hedenström A (2009) PIV-based investigation of animal flight. Exp Fluids 46: 749–763.

[51] Anderson JD Jr. (2001) Fundamentals of Aerodynamics, 3rd Ed., McGraw-Hill Higher Education.

[52] Videler J, Kamermans P (1985) Differences between upstroke and downstroke in swimming dolphins. J Exp Biol 119: 265–274. 4093758 10.1242/jeb.119.1.265

